# Alveolar adenoma of the lung: unusual diagnosis of a lesion positive on PET scan. A case report

**DOI:** 10.1186/1749-8090-7-1

**Published:** 2012-01-03

**Authors:** Mario Nosotti, Paolo Mendogni, Lorenzo Rosso, Davide Tosi, Alessandro Palleschi, Maria Basciu, Luigi Santambrogio, Stefano Ferrero

**Affiliations:** 1 Thoracic Surgery and Lung Transplantation Unit, Fondazione IRCCS Cà Granda Ospedale Maggiore Policlinico, University of Milan, Milan, Italy; 2 Pathology Department, Fondazione IRCCS Ca' Granda Ospedale Maggiore Policlinico, University of Milan, Milan, Italy

**Keywords:** Alveolar adenoma, Lobectomy, Lung, Positron emission tomography (PET)

## Abstract

The authors report a clinical case of alveolar adenoma presenting as a solitary pulmonary nodule which was positive to PET and deeply located in the lung. Few cases of alveolar adenomas have been reported in literature; these lesions are considered pulmonary neoplasms with benign behaviour, usually presenting as a peripheral or subpleural coin lesion; the PET activities of such neoplasms were unknown.

The present clinical case was singular for the deep location of the nodule and its tight adhesion to left inferior pulmonary vein requiring a lobectomy. In addition, alveolar adenoma PET behaviour has been reported as light positivity.

## Background

Alveolar adenoma is a rare lung neoplasm and few cases have been reported in literature. Such lesions are considered as benign neoplasm and no recurrences have been described. These neoplasms are usually located peripherally in the lung and their PET activities are unknown.

We report the first case of alveolar adenoma positive on PET scan, deeply located in left lower lobe.

### Case presentation

A 54-year-old, non-smoker woman presented to her primary care physician for chronic, non-productive cough and mild dyspnoea. Her past medical history included partial gastrectomy for gastric ulcer, colecystectomy, dyslipidemia and gastric reflux disease. She also reported a previous occupational exposure to benzene.

**Figure 1:**
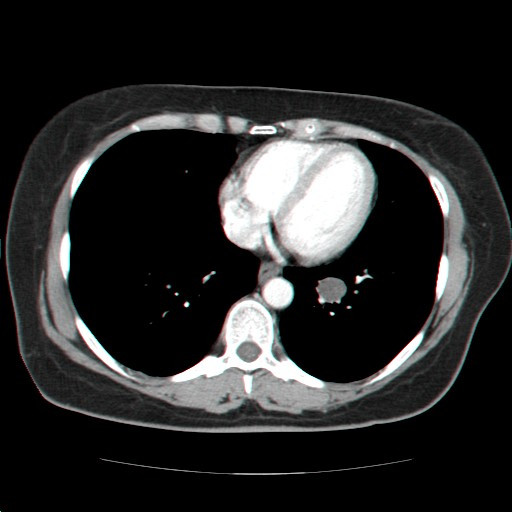
**Axial contrast enhanced CT scan of the chest reveales the presence of a smooth edged 18 mm pulmonary lesion in the left inferior hilum**.

Clinical examination was unremarkable and pulmonary function was normal. The chest X-ray and the subsequent enhanced chest computed tomography (CT) scan revealed the presence of a well-circumscribed, ovular, smooth edged, dense pulmonary lesion in the left inferior hilum (Figure 1). The diameter of the nodule was approximately 18 mm, with no contrast enhancement. No other significant abnormalities were detected, in particular there was neither pleural effusion nor mediastinal adenopathy. The patient underwent a fluorine-18-fluorodeoxyglucose Positron Emission Tomography (PET) scan, revealing minor uptake in the left lower lobe nodule with a Standardized Uptake Value of 1.06. The brain and the abdomen CT scans were negative for metastatic localizations.

In order to exclude a malignancy, a fibreoptic bronchoscopy with bronchial washing and a percutaneous CT-guided fine needle aspiration biopsy were performed. Both the examinations resulted negative for malignant disease.

Notwithstanding the negative results of the cytological investigations, the PET finding and the contiguity to the hilar structures convinced the authors to remove the pulmonary nodule. Therefore, a minimally invasive muscle-sparing lateral thoracotomy was performed in order to biopsy the pulmonary lesion. The intraoperative histological examination by frozen section suggested a cystic, well-delineated lesion without sign of malignant disease; however, the finding had to be confirmed by the definitive histology obtained by paraffin block.

Considering the nodule localization (inseparable from the inferior pulmonary vein) and the intraoperative histological finding, we decided for a left lower lobectomy. The surgical procedure and the postoperative course were uneventful. The patient has been discharged 5 days after the operation in good clinical condition; after one year, she is in good health and the chest CT scan doesn't reveal any sign of recurrence.

The resected lung segment contained a well-defined yellowish, round mass, with a diameter of approximately 2 cm, with a tight adhesion to the wall of the adjacent left inferior vein.

**Figure 2:**
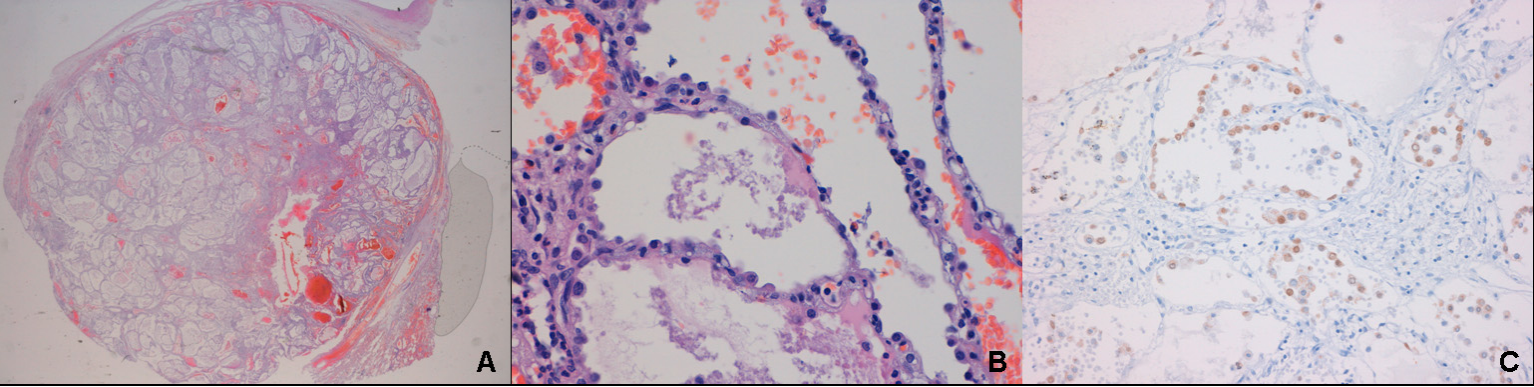
**Histolpathological findings.** Figure 2A and B: Haematoxilin-eosin stain of the lesion at 4x and 10x original magnification respectively. Figure 2C: Strong immunoreactivity in epithelial cuboidal cells for TTF-1 antibody (10x original magnification).

Histologically, the mass was well demarcated (Figure 2a) and composed of a network of cystic spaces lined with simple epithelial cuboidal type II pneumocytes cells without atypia that contain stroma ranging from thin inconspicuous strands of connective tissue to broad spectrum of spindle cells sometimes in a myxoid matrix (Figure 2b). Neither mitotic figures nor necrosis were detectable. The immunohistochemical profile in the epithelial component was positive for thyroid transcription factor (TTF-1) (Figure 2c) and negative for myogenin, surfactant and cytokeratins. The mesenchimal spindle cells components were unreactive for CD34, CD68 and desmin. The proliferative activity was assessed by Ki-67 and was unremarkable in both cell types.

Due to the morphological apparence and immunohistochemical findings of the lesion the final diagnosis was alveolar adenoma of the lung.

## Discussion

Alveolar adenoma is a very rare and unusual pulmonary neoplasm first described in 1986 by Yousem and Hochhololzer [1]. Although the exact number of case is unknown since these neoplasm have been confused with similar benign lesion, there are few cases reported. Only 29 cases were descibed in English medical literature [2,4,5] and almost all of them are peripheral and subpleural coin lesion, like a solitary pulmonary nodule. One reported case was localized near lung hilum^3 ^and another one was a multiseptated huge cystic mass^5^. Moreover, a case of simultaneous multiple localizations was also reported [6]. There is a predominance of middle aged female (2:1); usually the lesion is incidentally discovered on x-ray and the middle or the lower lobes are the preferred localizations. Commonly such neoplasm has a benign behaviour and there are no recurrences reported after the surgery.

The cytological preoperative diagnosis is surely difficult and has been never reported; intraoperative frozen section is troublesome too. Majors histological features of alveolar adenoma are a well circumscribed unencapsulated cystic mass composed by cuboidal epithelial cells immunoreactive to TTF-1 antibody without atypia surrounded by a myxoid and collagenous interstitium, in which elongated, plump, cells are usually detectable [7].

The origin and histogenesis of the neoplasm are unknown. This rare neoplasm should entered in differential diagnosis of solitary pulmonary nodule, whether in benign (e.g. slerosing hemangioma, papillary adenoma, amartochondroma) or in malignant tumors (e.g. broncho-alveolar carcinoma, atypical adenomatous hyperplasia).

The case we experienced is unique for its hilar presentation and its isotope uptake on PET scan. This is the first case reported with a positive PET scan considering that, to the authors' knowledge, the only PET scan reported in a clinical case with alveolar adenoma resulted negative [8]. Taking into account the benign behaviour of the alveolar adenoma, the faint uptake of the fluorodeoxyglucose in our patient is expected. PET scan is a part of our usual work up in patient with solitary pulmonary nodule; in this specific case the faint uptake should be useful during follow up.

In conclusion, the authors suggest considering alveolar adenoma as possible diagnosis in cases with light PET positive pulmonary nodule.

## Consent

Written informed consent was obtained from the patient for publication of this case report and accompanying images. A copy of written consent is available for review by the Editor-in-Chief of this journal.

## Competing interests

The authors declare that they have no competing interests.

## Authors' contributions

MN wrote the article and participated in the surgical operation. PM and AP collected data and contributed to the pre- and postoperative patient's management, LR participated in the design of the study, DT participated in the surgical operation and collected data. MB e SF carried out the histopathological diagnosis. LS performed the surgical operation and revised manuscript. SF helped in the draft of the study and revised the manuscript. All the authors approved the final version of the manuscript.
